# Interaction of tumor cells and astrocytes promotes breast cancer brain metastases through TGF-β2/ANGPTL4 axes

**DOI:** 10.1038/s41698-019-0094-1

**Published:** 2019-10-03

**Authors:** Xianghui Gong, Zhimin Hou, Michael P. Endsley, Emily I. Gronseth, Kevin R. Rarick, Julie M. Jorns, Qiuhui Yang, Zhenggui Du, Ke Yan, Michelle L. Bordas, Jill Gershan, Parashar Deepak, Anjali Geethadevi, Pradeep Chaluvally-Raghavan, Yubo Fan, David R. Harder, Ramani Ramchandran, Ling Wang

**Affiliations:** 1 Department of Obstetrics & Gynecology, Medical College of Wisconsin, 8701 Watertown Plank Road, Milwaukee, WI 53226 USA; 2 Key Laboratory for Biomechanics and Mechanobiology of Chinese Education Ministry, School of Biological Science and Medical Engineering, Beihang University, Xue Yuan Road No. 37, Haidian District Beijing, 100083 P. R. China; 3 Division of Obstetrics & Gynecology, Tianjin Union Medical Center, 130 Jianyuan Dao, Honqiao District Tianjin, 300190 P. R. China; 4 Department of Pediatrics, Children’s Research Institute, Medical College of Wisconsin, 8701 Watertown Plank Road, Milwaukee, WI 53226 USA; 5 Department of Pathology, Medical College of Wisconsin, 8701 Watertown Plank Road, Milwaukee, WI 53226 USA; 6 Department of Breast Surgery, West China Hospital, Sichuan University, Chengdu, Sichuan 610041 P. R. China; 7 Section of Quantitative Health Sciences, Department of Pediatrics, Medical College of Wisconsin, 8701 Watertown Plank Road, Milwaukee, WI 53226 USA; 8 Beijing Advanced Innovation Center for Biomedical Engineering, Beihang University, Xue Yuan Road No. 37, Haidian District Beijing, 100083 P. R. China; 9 Department of Physiology, Medical College of Wisconsin, 8701 Watertown Plank Road, Milwaukee, WI 53226 USA; 10 Department of Cell Biology, Neurobiology and Anatomy, Medical College of Wisconsin, 8701 Watertown Plank Road, Milwaukee, WI 53226 USA

**Keywords:** Cancer microenvironment, Breast cancer, Cancer microenvironment, Breast cancer

## Abstract

Metastatic outcomes depend on the interactions of metastatic cells with a specific organ microenvironment. Our previous studies have shown that triple-negative breast cancer (TNBC) MDA-MB-231 cells passaged in astrocyte-conditioned medium (ACM) show proclivity to form brain metastases, but the underlying mechanism is unknown. The combination of microarray analysis, qPCR, and ELISA assay were carried out to demonstrate the ACM-induced expression of angiopoietin-like 4 (ANGPTL4) in TNBC cells. A stable *ANGPTL4*-knockdown MDA-MB-231 cell line was generated by *ANGPTL4* short-hairpin RNA (shRNA) and inoculated into mice via left ventricular injection to evaluate the role of ANGPTL4 in brain metastasis formation. The approaches of siRNA, neutralizing antibodies, inhibitors, and immunoprecipitation were used to demonstrate the involved signaling molecules. We first found that ACM-conditioned TNBC cells upregulated the expression of ANGPTL4, a secreted glycoprotein whose effect on tumor progression is known to be tumor microenvironment- and tumor-type dependent. Knockdown of ANGPTL4 in TNBC MDA-MB-231 cells with shRNA decreased ACM-induced tumor cell metastatic growth in the brain and attributed to survival in a mouse model. Furthermore, we identified that astrocytes produced transforming growth factor-beta 2 (TGF-β2), which in part is responsible for upregulation of ANGPTL4 expression in TNBC through induction of SMAD signaling. Moreover, we identified that tumor cells communicate with astrocytes, where tumor cell-derived interleukin-1 beta (IL-1β) and tumor necrosis factor alpha (TNF-α) increased the expression of TGF-β2 in astrocytes. Collectively, these findings indicate that the invading TNBC cells interact with astrocytes in the brain microenvironment that facilitates brain metastases of TNBC cells through a TGF-β2/ANGPTL4 axis. This provides groundwork to target ANGPTL4 as a treatment for breast cancer brain metastases.

## Introduction

The incidence of breast cancer brain metastasis (BM) has increased in recent years, particularly in the subpopulation of triple-negative breast cancer (TNBC) (estrogen receptor <ER>-/progesterone receptor <PR>-/human epidermal growth factor receptor 2 <Her2>-, ER-/PR-/Her2-).^1–3^ TNBC, which accounts for 15–25% of all breast cancers,^4,5^ has problematic diagnosis because patients with TNBC do not respond to hormone or HER2-targeted therapies due to the lack of expression of ER, PR, and HER2.^4^ Moreover, the exact pathologic process of BM in the TNBC subset is poorly understood up to now. These facts emphasize the clinical importance of BM in TNBC and provide an explanation for why patients with TNBC have the worst outcomes out of all breast cancer patients. Therefore, much effort is needed on this front to help address BM in TNBC.

Ultimately, the clinical outcomes of people with metastatic cancer greatly rely on the interactions of the metastatic tumor cells and the cells in the surrounding microenvironment of an organ.^6,7^ Astrocytes, a subtype of glial cells, are the most abundant cell type in the brain microenvironment. In breast cancer BM patients, reactive astrocytes accumulate around the metastatic foci;^8–10^ they confine brain metastases without infiltrating the lesion.^11^ Previous studies have found that reactive astrocytes participate in tumor progression and chemoresistance through direct physical contact and gap junctional communication with tumor cells in the brain.^12,13^ It has also been reported that paracrine signaling via cytokines, growth factors, or exosomes released by astrocytes facilitates tumor metastasis formation in the brain and affects blood–brain barrier permeability.^9,10,14,15^ On the other hand, the interaction between tumor cells and astrocytes may not be just synergistic. There was a report that brain-metastatic cells express high-level serpins to prevent astrocyte-generated plasmin and its metastasis-suppressive effects.^16^ Although these studies have provided strong evidence about the role of astrocyte tumor BM, the underlying mechanisms remain poorly understood.

We have previously shown that astrocyte-conditioned medium (ACM) facilitated metastasis formation of MDA-MB-231 cells in the brain.^17^ In this follow-up study, we further investigated the underlying mechanism for the ACM-induced BM phenotype. After conditioning TNBC cells in ACM, we found that ACM upregulated the expression of angiopoietin-like 4 (ANGPTL4) in TNBC cells. ANGPTL4 is a secreted glycoprotein whose effect on tumor progression is tumor-type- and microenvironment-dependent.^18–20^ It has been shown that transforming growth factor-beta 1 (TGF-β1) induced ANGPTL4 expression in breast cancer cells, which facilitated breast cancer cells to metastasize to the lung.^21^ Mouse astrocytes secrete TGF-β2,^22^ a homolog protein of TGF-β1.^23,24^ On this basis, we investigated how tumor cells and astrocytes interact to contribute to breast cancer BM through the TGF-β2/ANGPTL4 axis.

## Results

### ACM upregulates ANGPTL4 expression in TNBC cells

**Fig. 1:**
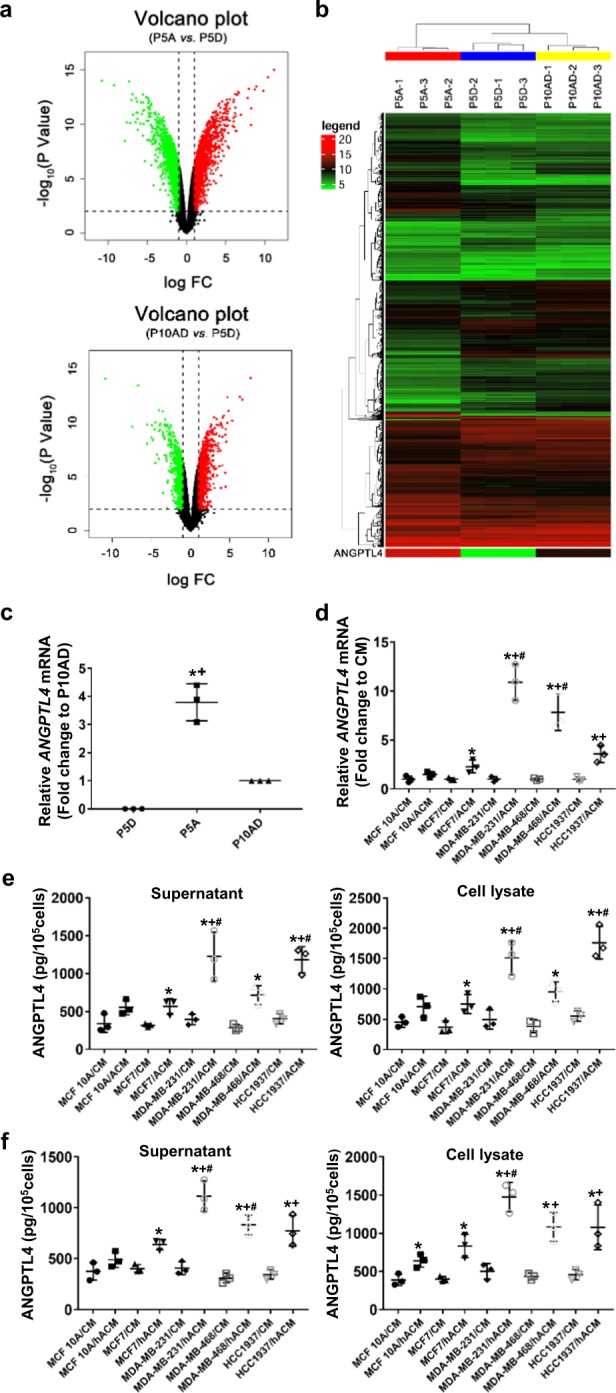
ACM upregulates ANGPTL4 expression in TNBC cells. **a** Volcano plots for differential ACM-responsive genes. The log-fold change relative to P5D is plotted on the *x*-axis (the downregulated genes represented by green points, the upregulated genes represented by red points) and the negative log10 *p*-value is plotted on the *y*-axis. The black, dashed line represents the *p*-value cutoff (0.01, Student’s *t*-test) and log FC cutoff (±1.0). **b** Heatmap of differential ACM-responsive gene expression. Heatmap of gene expression alterations seen across all probe sets, with triplicate samples per culture condition. Each column represents a single culture and each row represents a single gene. Red indicates overexpressed probe sets, whereas green indicates underexpressed probe sets. The top cluster diagram indicates the hierarchical clustering order of the individual samples analyzed. The right cluster diagram represents differentially expressed probes with *p* < 0.01 and FC > 2.0. As shown, ANGPTL4 is one of the top overexpressed genes. **c** qPCR of *ANGPTL4* transcripts in ACM-conditioned MDA-MB-231 cells. *p < 0.05 compared with P5D and ^+^*p* < 0.05 compared with P10AD by the two-sided Student’s *t*-test, *n* = 3. **d** qPCR of *ANGPTL4* transcripts in ACM-conditioned TNBC cells. *p < 0.05 compared with CM control, ^+^*p* < 0.05 compared with MCF-10A in ACM, and ^#^*p* < 0.05 compared with MCF-7 in ACM by the two-sided Student’s *t*-test. *n* = 3. **e** ELISA analysis of the expression of ANGPTL4 in supernatants and cell pellets of the ACM-conditioned TNBC cells. *p < 0.05 compared with CM control, ^+^*p* < 0.05 compared with MCF-10A in ACM, and ^#^*p* < 0.05 compared with MCF-7 in ACM by the two-sided Student’s *t*-test. *n* = 3. **f** ELISA analysis of ANGPTL4 in hACM-conditioned TNBC cells. *p < 0.05 compared with CM control, ^+^*p* < 0.05 compared with MCF-10A in hACM, and ^#^*p* < 0.05 compared with MCF-7 in hACM by the two-sided Student’s *t*-test. *n* = 3. From (**c**) to (**f**), CM: tumor cell culture medium; ACM: rat astrocyte-conditioned medium; hACM: human astrocyte-conditioned medium

The most intriguing finding from our earlier studies is that after passaging the MDA-MB-231 cells in ACM for five passages, the invasion competence of the tumor cell showed a sudden and rapid increase, which led to an increase in vitro invasion when compared with the cells passaged 1–4 times in ACM (fold change to cells passaged five times in DMEM: 1.9, 2.2, 6.8, 7.4, and 19.8).^17^ During inoculation of the passage 5 cells into mice through left ventricular injection, rapid and severe brain metastases were developed.^17^ To gain insight into the mechanism of the BM, we investigated whether the ACM affected gene expression by microarray expression analysis using MDA-MB-231/P5A, MDA-MB-231/P5D (control), and MDA-MB-231/P10AD (determining if the ACM elicited permanent or transient effects on the cells). Based on the data of the 15,453 gene-level probe sets, we sought for the genes with significant expression changes (*p* < 0.01 and FC > 2.0) in response to ACM. The distribution of genes according to the significance of their expression was represented in volcano plots (Fig. 1a). We also generated a hierarchical clustering heatmap across the nine samples under the three different cultures (Fig. 1b). Our results showed that the expression of 3676 genes was significantly changed with 1875 upregulated genes and 1801 downregulated genes in MDA-MB-231/P5A vs MDA-MB-231/P5D (Fig. 1a upper panel. Additional file 2 Table S1 for gene lists), and the expression of 1637 genes significantly changed with 922 upregulated genes and 715 downregulated genes in MDA-MB-231/P10AD vs MDA-MB-231/P5D (Fig. 1a lower panel. Additional file 2 Table S2 for gene lists). Of interest, clustering analyses across the nine samples under the three cultures grouped MDA-MB-231/P10AD and MDA-MB-231/P5D in the same cluster (Fig. 1b), which hints that the expression genes from MDA-MB-231/P10AD cells are more similarly relative to the genes from MDA-MB-231/P5D cells. Together, these results suggest that ACM may induce and maintain genetic alteration in MDA-MB-231 cells.

Based on our microarray analysis, *ANGPTL4* is one of the most upregulated genes. ANGPTL4 has been recently emerging as an important factor in tumor progression.^18,21,25^ Therefore, qPCR was performed to confirm the expression of *ANGPTL4* in the three cells (Fig. 1c). *ANGPTL4* expression was significantly higher in MDA-MB-231/P5A cells when compared with MDA-MB-231/P5D (*p* = 0.010 by the two-sided Student’s *t*-test) and MDA-MB-231/P10AD cells (*p* = 0.018 by the two-sided Student’s *t*-test), which suggests that ACM induced and maintained the expression of *ANGPTL4* in MDA-MB-231 cells.

To examine whether ACM upregulates *ANGPTL4* expression in other TNBC cells, TNBC MDA-MB-231, MDA-MB-468, HCC1937 cells, breast cancer estrogen receptor-positive cells (MCF-7), and immortalized breast epithelial cells (MCF-10A) were sequentially passaged in ACM for five passages. Cells passaged in the corresponding cell culture media (CM) were used as control. Gene expression was then analyzed by qPCR. Our data showed that basal expression of *ANGPTL4* in cells cultured in media (CM) was similar (Additional file 3: Fig. S1a). However, after being passaged in ACM, the expression of *ANGPTL4* significantly increased in all tumor cells compared with CM control (*p* < 0.05 in MCF-7 and *p* < 0.005 in TNBC cells by the two-sided Student’s *t*-test, Fig. 1d). Moreover, the expression of *ANGPTL4* significantly increased in all TNBC cells compared with MCF-10A cells (*p* < 0.01 by the two-sided Student’s *t*-test, Fig. 1d), as well as in MDA-MB-231 and MDA-MB-468 cells compared with MCF-7 cells (*p* < 0.005 by the two-sided Student’s *t*-test, Fig. 1d). Furthermore, we examined protein expression of ANGPTL4 in cell lysates and culture supernatants by ELISA (Fig. 1e). We found that the ANGPTL4 protein expression significantly increased in all tumor cells when compared with CM control (*p* < 0.05 by the two-sided Student’s *t*-test), and MDA-MB-231 and HCC1937 cells in ACM expressed significantly higher ANGPTL4 than MCF-10A and MCF-7 cells in ACM (*p* < 0.05 by the two-sided Student’s *t*-test). The expression of ANGPTL4 was also examined with cells passaged in human-derived astrocyte-conditioned medium (hACM), which revealed similar results as cells cultured in ACM (Fig. 1f and Additional file 3: Fig. S1b). These data suggest that astrocytes secrete factors that upregulate ANGPTL4 expression especially in TNBC cells.

### ANGPTL4 contributes to the metastatic lesions of MDA-MB-231 cells in the brain

**Fig. 2:**
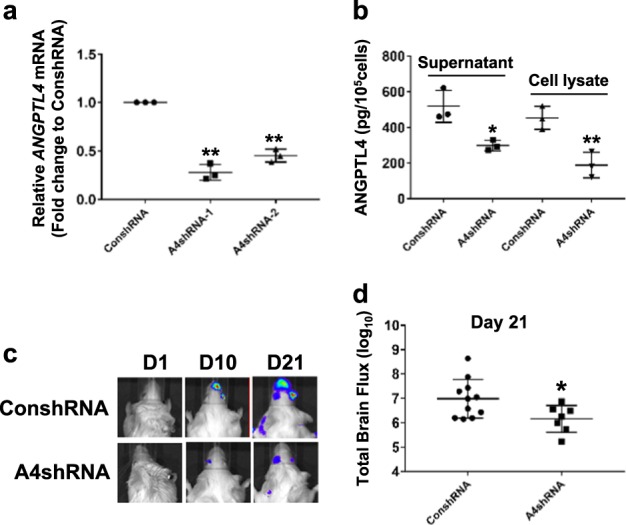
ANGPTL4 contributes to the brain metastases of MDA-MB-231 cells. **a** *ANGPTL4* s6hRNA decreased mRNA expression level of *ANGPTL4* in MDA-MB-231 cells. MDA-MB-231 cells were transfected with *ANGPTL4* shRNA-1 and shRNA-2, respectively, or non-targeting control shRNA and further used for qPCR analysis for *ANGPTL4* expression. **p < 0.01 compared with the control group by the two-sided Student’s *t*-test. **b** Effects of *ANGPTL4* knockdown on ANGPTL4 protein expression in MDA-MB-231 cells. MDA-MB-231 cells transfected with A4shRNA-1 (A4shRNA) or control shRNA (ConshRNA) were used for ELISA analysis of ANGPTL4 expression. *p < 0.05 and **p < 0.01 compared with the control group by the two-sided Student’s *t*-test. **c**, **d** Role of ANGPTL4 in brain lesions of MDA-MB-231 cells. A4shRNA/ACM (*n* = 7) and ConshRNA/ACM MDA-MB-231 (*n* = 11) cells were injected into the left ventricle of nude mice. Tumor lesions were measured by bioluminescent imaging. **c** Representative brain metastasis tumor images. **d** Total brain flux at 21 days post injection. *p = 0.029 by the two-sided Student’s *t*-test comparing A4shRNA/ACM (mean 2.51e + 6, SD 2.56e + 6) with ConshRNA/ACM (mean 5.35e + 7, SD 1.29e + 8)

Next, we investigated the role of ANGPTL4 in in vivo BM of TNBC cells. With four unique 29-mer shRNA constructs, two stable *ANGPTL4*-knockdown MDA-MB-231 cell lines (A4-1shRNA and A4-2shRNA) were established (*p* < 0.01 by the two-sided Student’s *t*-test, Fig. 2a). Due to the higher knockdown level in the MDA-MB-231/A4-1shRNA cell line, it was simply named MDA-MB-231/A4shRNA and selected for the in vivo experiment after confirming its protein expression level (*p* < 0.05 by the two-sided Student’s *t*-test, Fig. 2b). Cells transfected with scrambled shRNA were used as control (MDA-MB-231/ConshRNA).

The MDA-MB-231/A4shRNA and MDA-MB-231/ConshRNA cells were passaged in ACM for five passages. After confirming the expression level of ANGPTL4 (Additional file 3: Fig. S2a), these cells were injected into 6–8-week-old male CB17/SCID mice through the left ventricle, and bioluminescence imaging was performed to visualize brain-metastatic lesions (Additional file 3: Fig. S2b, c and Fig. 2c). Brain metastases were evaluated. Total flux was used to monitor tumor growth. We found that the total brain flux at day 21 was significantly lower in A4shRNA/ACM compared with ConshRNA/ACM (*p* = 0.029 by the two-sided Student’s *t*-test, Fig. 2d). On the last day of measurement (either the day of death or day 42 for those mice that survived), total brain flux had no statistically significant difference between the two groups (*p* = 0.31 by the two-sided Student’s *t*-test. Additional file 3: Fig. S2d). When comparing the time to BM between the two groups, we also did not see significant differences (*p* = 0.36 by the log-rank test. Additional file 3: Fig. S2e). However, if we compared the fold change of ConshRNA/ACM with respect to A4shRNA/ACM on the last day, total flux was more than two fold, and BM incidence was about 1.4-fold (Additional file 3: Fig. S2c, e). Moreover, the survival of mice was about 3.14-fold compared with MB-231/A4shRNA and MDA-MB-231/ConshRNA cells (4 out of 7 and 2 out of 11. Additional file 3: Fig. S2c). Together, these results suggest that knockdown of ANGPTL4 decreases MDA-MB-231 cell metastatic growth in the brain.

### TGF-β2 derived from astrocytes upregulates the expression of ANGPTL4 in TNBC cells

**Fig. 3:**
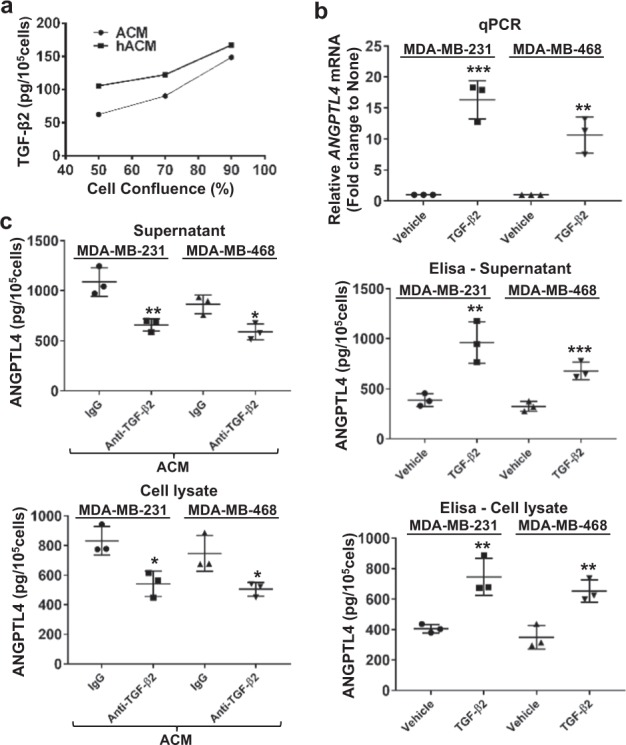
Astrocyte-secreted TGF-β2 induces ANGPTL4 expression in TNBC cells. **a** Rat and human astrocytes produce TGF-β2. TGF-β2 was quantified by ELISA analysis in both rat and human ACM. **b** TGF-β2-induced *ANGPTL4* expression in MDA-MB-231 and MDA-MB-468 cells. Cells were treated with vehicle or TGF-β2 (5 ng/ml) and ANGPTL4 expression was quantified in both mRNA and protein by qPCR and ELISA, respectively. **p < 0.01 and ***p < 0.005 compared with vehicle by the two-sided Student’s *t*-test, *n* = 3. **c** Astrocyte-derived TGF**-**β2 was involved in the ANGPTL4 induction in TNBC cells. ACM with or without TGF-β2 depletion was used to culture MDA-MB-231 and MDA-MB-468 cells, and analyzed by ELISA to quantify ANGPTL4 expression. *p < 0.05 and **p < 0.01 compared with ACM by the two-sided Student’s *t*-test. *n* = 3. ACM: rat astrocyte-conditioned medium; hACM: human astrocyte-conditioned medium

TGF-β1 has been shown to induce ANGPTL4 expression in TNBC cells, leading to pulmonary metastases.^21^ There are reports that TGF-β2, but not TGF-β1, is present in the culture supernatants of mouse astrocytes,^22^ and cultured human adult astrocytes produce biologically active TGF-β2 but only minimal TGF-β1.^26^ Because TGF-β2 and TGF-β1 are highly homologous,^23,24^ we investigated whether astrocyte-secreted TGF-β2 can regulate ANGPTL4 expression. TGF-β2 was detected in both ACM and hACM by ELISA (Fig. 3a). We then sought to examine whether astrocyte-secreted TGF-β2 induces ANGPTL4 expression in TNBC cells. To do so, we first examined whether the exogenous addition of TGF-β2 could induce ANGPTL4 expression in TNBC cells. MDA-MB-231 and MDA-MB-468 cells treated with human recombinant TGF-β2 (5 ng/ml, Pepro Tech, Inc.) showed that TGF-β2 upregulated both RNA and protein expression of ANGPTL4 (*p* < 0.01 by the two-sided Student’s *t*-test, Fig. 3b). We then analyzed whether astrocyte-secreted TGF-β2 was involved in the induction of ANGPTL4 in TNBC cells. TGF-β2- depleted ACM was used to culture the TNBC cells for five passages. A reduction of ANGPTL4 in supernatants and cell lysates from cells cultured in TGF-β2-depleted ACM was observed when compared with cells cultured in regular ACM (*p* < 0.05 by the two-sided Student’s *t*-test, Fig. 3c). Collectively, these data suggest that astrocytes secrete TGF-β2, which upregulates the expression of ANGPTL4 in TNBC cells.

### TGF-β2 upregulates the expression of ANGPTL4 in TNBC cells through canonical TGF-β pathway

**Fig. 4:**
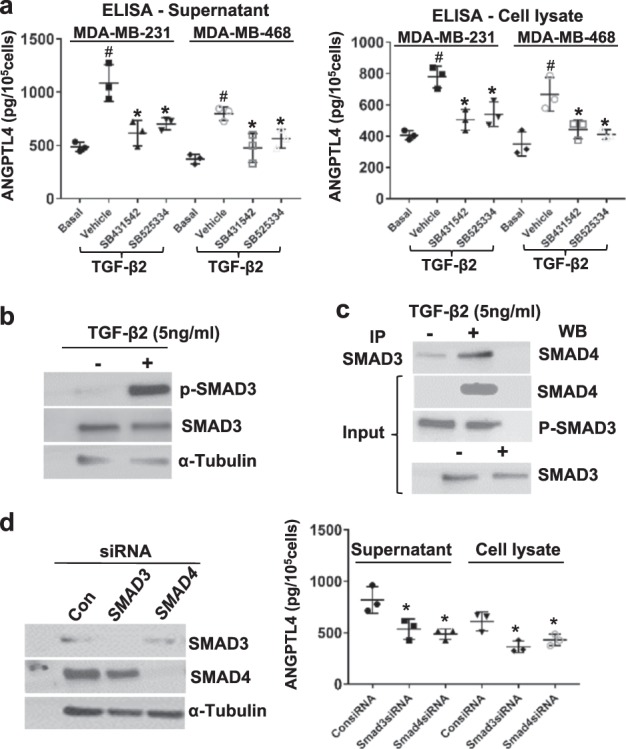
TGF-β2 induces ANGPTL4 expression through the TGFβR/SMAD signaling pathway. **a** TGFβR mediated ANGPTL4 expression in TNBC cells. TNBC cells were treated with vehicle or SB431542 or SB525334, and then stimulated with 5 ng/ml TGF-β2. The resultant supernatants and cell lysates were analyzed by ELISA to quantity ANGPTL4 expression. ^#^*p* < 0.01 compared with basal, *p < 0.05 compared with vehicle by the two-sided Student’s *t*-test, *n* = 3. **b** TGF-β2 activated SMAD3. Immunoblot (IB) was performed to examine the phosphorylation of SMAD3 upon TGF-β2 (5 ng/ml) induction. **c** TGF-β2 attributed to SMAD3/SMAD4 complex formation in MDA-MB-231 cells. Immunoprecipitation (IP) followed by IB experiments was performed to examine the SMAD3/SMAD4 complex in MDA-MB-231 cells upon TGF-β2 (5 ng/ml) induction. **d** SMAD3 and SMAD4 mediated TGF-β2-induced ANGPTL4 expression in MDA-MB-231 cells. The expression of *SMAD3* and *SMAD4* in MDA-MB-231 cells were knocked down by siRNAs (left panel), and the resultant supernatants and the cell lysates were analyzed by ELISA to quantity ANGPTL4 expression (right panel). *p < 0.05 compared with control siRNA-treated cells by the two-sided Student’s *t*-test. *n* = 3

On the above basis, the upstream signaling pathway involved in the induction of ANGPTL4 by TGF-β2 in TNBC cells was investigated. It is well known that TGF-β signals via its transmembrane serine/threonine kinase receptors. Binding of TGF-β to TGF-βR2 recruits TGF-βR1 into the receptor complex, resulting in the phosphorylation of TGF-βR1 to transmit the signal to downstream signaling molecules.^27,28^ To examine whether TGF-βR is involved in the ANGPTL4 induction, MDA-MB-231 and MDA-MB-468 cells were first treated with TGF-βR1 inhibitors, SB431542 and SB525334 (Selleck Chemicals), respectively, and then stimulated with TGF-β2 (5 ng/ml). The results showed that blocking TGF-βR1 function reduced TGF-β2-induced ANGPTL4 expression in TNBC cells (*p* < 0.05 by the two-sided Student’s *t*-test, Fig. 4a). These data suggest that TGF-βR1 mediates the induction of ANGPTL4 by TGF-β2.

Phospho-TGF-βR1 activates transcription factors SMAD2 and SMAD3 via phosphorylation. Phosphorylated SMAD2/3 subsequently forms a complex with co-SMAD (SMAD4) and enters the nucleus to regulate gene transcription.^27^ Thus, SMAD participation in the induction of ANGPTL4 by TGF-β2 was examined. After treating MDA-MB-231 cells with TGF-β2 (5 ng/ml) for 6, 24, and 48 h, the expression of phospho-SMAD3 was increased (Fig. 4b shows the expression of ANGPTL4 at 6 h). The SMAD3/SMAD4 complex formation was induced after 24-h treatment with TGF-β2 in MDA-MB-231 cells (Fig. 4c). The involvement of SMAD3/SMAD4 signaling in TGF-β2-induced ANGPTL4 expression was further verified when knockdown of SMAD3/SMAD4 reduced ANGPTL4 expression in MDA-MB-231 cells (*p* < 0.05 by the two-sided Student’s *t*-test, Fig. 4d). Collectively, these data suggest that the canonical TGF-β/SMAD pathway mediates TGF-β2-induced ANGPTL4 expression in TNBC cells.

### TNBC cell-derived IL-1β and TNF-α enhance the expression of TGF-β2 in astrocytes

**Fig. 5:**
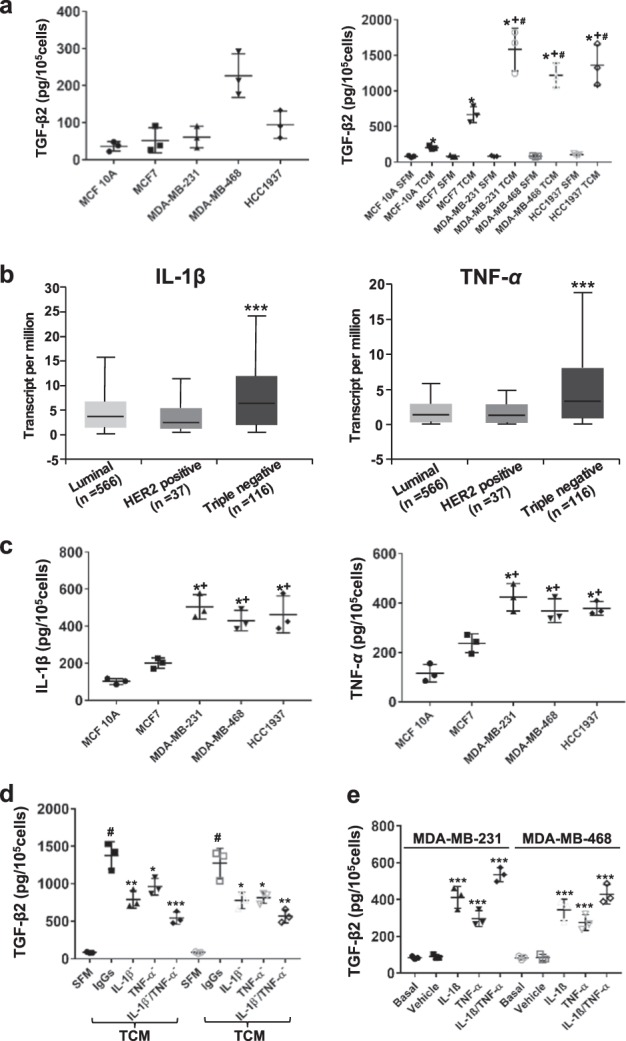
TNBC cell-derived IL-1β and TNF-α enhance the expression of TGF-β2 in astrocytes. **a** Astrocyte-derived TGF-β2 was increased by tumor cell-conditioned medium (TCM) from TNBC cells. Quantity of basal TGF-β2 expressions in TCM (left panel); TCM was added to culture astrocytes, and the resultant culture supernatants were analyzed by ELISA to quantify TGF-β2 expression (right panel). Astrocytes cultured in serum-free breast cancer cell line media (SFM) that was not preconditioned by tumor cells were used as control. *p < 0.05 compared with astrocytes in TCM with SFM; ^+^*p* < 0.05 compared with MCF-10A in TCM and ^#^*p* < 0.05 compared with MCF-7 in TCM by the two-sided Student’s *t*-test. *n* = 3. **b** Mean centralized the mRNA of IL**-**1β and TNF-α in three subtypes of breast cancer. Data from TCGA 719 breast cancer cohort. ***p < 0.005 compared with luminal and Her2 + breast cancers by the two-sided Student’s *t*-test. **c** TNBC cells produced IL-1β and TNF-α. TCM from different cells was analyzed by ELISA to quantity the levels of IL-1β (left) and TNF-α (right), respectively. *p < 0.05 compared with MCF-10A in TCM and ^+^*p* < 0.05 compared with MCF-7 in TCM by the two-sided Student^’^s *t*-test. *n* = 3. **d** TNBC cell-derived IL-1β and TNF-α enhanced TGF-β2 expression in astrocytes. TCM treated with or without neutralizing anti-IL-1β or/and anti-TNF-α antibodies was used to culture astrocytes, and the resultant media were analyzed by ELISA to quantity TGF-β2 expression. SFM: serum-free medium. TCM: tumor cell-conditioned medium. ^#^*p* < 0.01 compared with SFM, *p < 0.05, **p < 0.01, and ***p < 0.005 compared with TCM by the two-sided Student’s *t*-test. *n* = 3. **e** IL-1β and TNF-α induced TGF-β2 expression in astrocytes. Astrocytes treated with vehicle or IL-1β (10 ng/ml, 6 h) or/and TNF-α (10 ng/ml, 6 h) were analyzed by ELISA to quantify TGF-β2 expression. *p < 0.05, **p < 0.01, and ***p < 0.005 compared with vehicle by the two-sided Student’s *t*-test. *n* = 3

Astrocytes are typically in an inactive, quiescent state in vivo. However, in response to brain injury, stroke, or brain cancer pathologic conditions, astrocytes become activated, a process named reactive gliosis, which is associated with profound changes in gene expression.^29–32^ Although astrocytes also tend to be very reactive in cell culture,^33^ astrocyte activity is a highly heterogeneous state depending on the specific injury.^34^ Thus, whether TNBC cells affect the TGF-β2 expression by astrocytes was examined. Following the demonstration of the low TGF-β2 expression level in tumor-conditioned medium (TCM) from the cultures of TNBC MDA-MB-231, MDA-MB-468, and HCC1937 cells in serum-free breast cancer cell line media (Fig. 5a left panel), the media were used in culture with astrocytes, respectively. Astrocytes cultured in the corresponding serum-free tumor cell culture medium (SFM) that was not preconditioned by tumor cells was used as control. After 24 h in the TCM, the amount of TGF-β2 produced by the astrocytes was measured by ELISA. A significant increase in TGF-β2 in the resultant astrocyte culture media was observed compared with astrocytes in TCM with those in SFM (*p* < 0.005 in MCF-10A, *p* = 0.0001 in MCF-7, and *p* < 0.0001 in TNBC cells by the two-sided Student’s *t*-test, Fig. 5a right panel), as well as compared with astrocytes in TCM from TNBC cells with those in non-TNBC cells (*p* < 0.0005 in MCF-10A and *p* < 0.05 in MCF-7 by the two-sided Student’s *t*-test, Fig. 5a right panel). This suggests that breast tumor cells, especially TNBC cells secrete factors into the TCM that increase the expression of TGF-β2 in astrocytes.

It has been reported that IL-1β and TNF-α increased the expression of TGF-β2 in human retinal pigment epithelial cells.^35^ Both IL-1β and TNF-α are highly expressed in invasive ductal carcinoma (IDC).^36^ Also, 90% of TNBCs are considered to be IDC.^37^ Therefore, TNBC cells may induce TGF-β2 expression in astrocytes through IL-1β and TNF-α produced by TNBC cells. To test this possibility, we first accessed transcriptome data of different breast cancer subtypes from TCGA. By analyzing the data from 719 breast cancer samples in which the luminal is 566, Her2 + is 37, and TNBC is 116, we found that the expression of IL-1β and TNF-α showed a significant increase in TNBC compared with luminal and Her2 + breast cancers (*p* < 0.005 by the two-sided Student’s *t*-test, Fig. 5b). Then, the significant higher expression level of IL-1β and TNF-α from TNBC cells compared with non-TNBC cells was confirmed by ELISA (*p* < 0.005 for IL-1β and *p* < 0.05 for TNF-α by the two-sided Student’s *t*-test, Fig. 5c). After that, TNF-α and IL-1β (separately and together) within the TCM were first neutralized by using neutralizing antibodies, and the resultant TCMs were then applied to astrocyte cultures. The results showed that neutralization of the two cytokines, both separately and together, led to decreased TGF-β2 expression by astrocytes (*p* < 0.05 by the two-sided Student’s *t*-test, Fig. 5d). In addition, after treating astrocytes with human recombinant IL-1β (10 ng/ml, R&D Systems) and TNF-α (10 ng/ml, R&D Systems) for 24 h in serum-free media, the resultant culture supernatant revealed higher expression levels of TGF-β2 than the culture supernatant from non-treated astrocytes (*p* < 0.005 by the two-sided Student’s *t*-test, Fig. 5e). Together, these results suggest that TNBC cell-secreted IL-1β and TNF-α increase the expression of TGF-β2 in astrocytes.

## Discussion

The development of BM occurs through multiple steps. Colonization and growth of metastatic tumor cells in the brain parenchyma is the most complex and rate-limiting phase of metastasis, which is dependent on the cross-talk between tumor cells and the brain microenvironment.^38,39^ Studies from human breast cancer–brain xenograft mouse models and clinical cases found that brain-metastatic colonization is accompanied by an extensive response in the surrounding brain tissue, recruiting large numbers of reactive astrocytes, which further associate with metastatic brain lesions.^8,9,11^ Our published data have shown that the ACM-passaged MDA-MB-231 cells demonstrated a higher incidence of tumor cell seeding in the brain and developing brain lesions in a mouse model compared with MDA-MB-231 cells passaged in DMEM.^17^ A recent study also reported that ACM induced cell elongation and promoted actin stress fiber organization in metastatic breast cancer cells.^40^ These observations suggest that astrocytes may derive cytokine and growth factors through paracrine signaling to promote the ability of breast cancer cells to seed and grow in the brain. In this study, we identified that astrocytes secrete TGF-β2 to upregulate ANGPTL4 expression in TNBC cells, which contributed to tumor cell brain lesions.

ANGPTL4 is a secreted protein belonging to a family of angiopoietin-like proteins, which has been known for its role as an adipokine involved in the regulation of lipid and glucose metabolism.^41^ It has been reported that upregulation of ANGPTL4 was observed in various types of human cancers, such as colorectal cancer and breast cancer.^18,21,25^ Elevated ANGPTL4 expression in these cancers correlates to enhanced metastasis, tumorigenesis, and a poor prognosis, while downregulation of ANGPTL4 leads to the opposite effect.^18,19,21,25^ However, studies have also shown that increased ANGPTL4 expression inhibits melanoma, lung, and colorectal tumor growth, metastasis, and angiogenesis.^20,42^ Thus, the expression and the role of ANGPTL4 in tumors appears to be context- and tumor-type dependent, which suggests the clinical importance of ANGPTL4 in tumor biology. As for breast cancer BM, a previous study has revealed that *ANGPTL4* is one of 17 genes within the breast cancer brain metastasis gene set (BrMS) whose expression was correlated with brain relapse in clinically annotated breast tumors and resembled the expression profile of brain-metastatic-derived cells from a mouse model.^43^ In this study, we found that knockdown of *ANGPTL4* in MDA-MB-231 cells significantly reduced the ability of these tumor cells to seed and grow in the brain at 21 days post injection. The significance at later time points may have been slightly skewed by the fact that murine Angptl4 is highly homologous to human ANGPTL4. Therefore, Angptl4 produced from other, non-tumor cell tissues in the mouse, may cause more variation in the results. For example, adipocyte-derived ANGPTL4 drives disease progression under obese conditions, thus demonstrating that ANGPTL4 produced from other cell types may still promote tumor progression.^44^ However, this finding is a direct evidence for the tumor-promoting role of ANGPTL4 in breast cancer BM, which provides groundwork to warrant further investigation into targeting ANGPTL4 as a treatment for breast cancer BM.

By focusing on TGF-β2, a homolog of TGF-β1, this study identified that the induction of ANGPTL4 in TNBC cells is through astrocyte-derived TGF-β2 (Fig. 3). However, TGF-β2 is only partially responsible for the ANGPTL4 induction by ACM, as tumor cell ANGPTL4 induction in the full ACM was about 1.6-fold compared with that in the TGF-β2-depleted ACM, whereas it was about threefold compared with that in DMEM. Thus, the ANGPTL4 expression from the cells passaged in the TGF-β2-depleted ACM is almost twofold of the cells passaged in DMEM. This indicates that TGF-β2 is just responsible for about 50% of ACM-induced ANGPTL4 upregulation. Considering the hundreds of cytokines and growth factors in ACM, this 50% of effect makes TGF-β2 one of the major factors derived by astrocytes that regulate the expression of ANGPTL4 in brain-metastatic breast cancer cells. Furthermore, this study identified that TGF-β2 induces ANGPTL4 expression through TGF-βR to activate Smad3, which leads to the formation of Smad3/Smad4 complex to upregulate the expression of ANGPTL4. SMAD4 knockdown reduced TGF-β2-mediated induction of ANGPTL4 expression, suggesting its involvement in metastasis. While SMAD4 is generally considered a tumor-suppressor protein, it has also been demonstrated to be involved in metastasis of breast cancer.^45^ Specifically, SMAD4 knockdown in MDA-MB-231 cells significantly inhibited bone metastasis when injected into nude mice.^45^ In agreement with this study, our findings suggest that SMAD4 involvement in tumor progression may depend on a variety of factors including tumor microenvironment. Another important fact that we observed in this study is that the upregulated ANGPTL4 level by ACM was decreased after re-passaging the cells in the DMEM, which shows that the regulation of ANGPTL4 is astrocyte dependent and is reversible.

In response to specific pathological conditions, astrocytes become activated by differently changing their gene expression in the process of reactive gliosis. In brain-metastatic melanoma, astrocytes were able to activate and produce more heparanase to promote the brain colonization of melanoma cells.^46^ In response to HER2-transfected MDA-MB-231 breast cancer cells, astrocytes activated and expressed phosphorylated p751-PDGFRβ, which in turn contributed to the brain-metastatic growth of the tumor cells.^47^ Brain-metastatic cells induce and maintain STAT3 activity in reactive astrocytes surrounding metastatic lesions that are required for BM.^11^ This astrocyte characteristic challenges our current approach of using ACM collected from in vitro cell culture to investigate the astrocyte-derived factors. Therefore, to confirm the experimental results by using the in vitro cell culture-acquired ACM, we cultured astrocytes with TCM from three different TNBC cell lines. Our data identified that in response to tumor cell-derived IL-1β and TNF-α, astrocytes not only continued to express TGF-β2, but the expression increased significantly. This also suggests that the interaction of astrocytes and breast cancer cells in brain microenvironment may lead to a positive-feedback loop and thus stronger tumor growth-promoting effects.

**Fig. 6:**
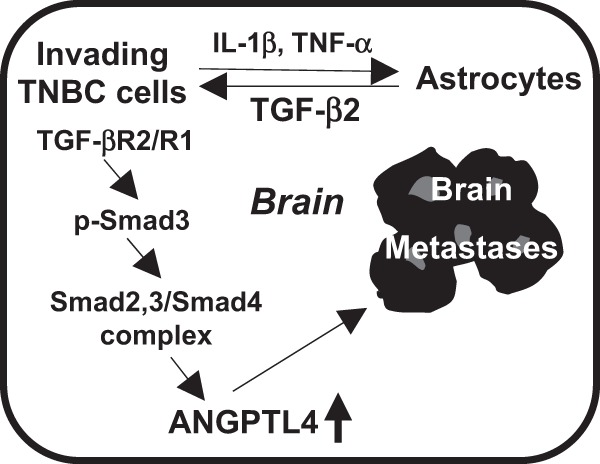
Working hypothesis. Invading tumor cells derived IL-1β and TNF-α that upregulated the expression of TGF-β2 in astrocytes. The TGF-β2 then through TGF-βR and SMAD signaling induced the expression of ANGPTL4 in tumor cells to promote brain lesions of the tumor cells

Taken together (Fig. 6), this study has shown for the first time that astrocytes produce more TGF-β2 in response to tumor cell-derived IL-1β and TNF-α. When exposed to ACM from astrocytes, the TGF-β2 induces TNBC cells to express more ANGPTL4 via the canonical TGF-β pathway. Expression of ANGPTL4 is associated with an increased ability of TNBC cells to seed and grow in the brain. This study aids in the understanding of how TNBC cells gain the abilities to thrive in the brain. However, questions remain, such as those related to the functional role of ANGPTL4 in BM and transcriptional regulation of *ANGPTL4*. One possible mechanism for ANGPTL4 facilitation of breast cancer brain metastasis is through disrupting vascular endothelial junctions, much like the mechanism for ANGPTL4 facilitation of breast cancer lung metastasis.^21^ Another potential mechanism includes ANGPTL4 binding to integrins β1 and β5. This binding hijacks the integrin/FAK-regulated pathway, thus conferring anoikis resistance in tumors, leading to increased tumor growth. In this model, astrocyte-mediated upregulation of ANGPTL4 in MDA-MB-231 cells allows for colonization in the brain microenvironment.^18^ Even so, this study has provided evidence for a novel target of ANGPTL4 to reduce breast cancer BM. Although targeting TGF-β is complex, as it is known to have roles in both inhibiting and promoting tumor growth, this research brings light to other parts of the complex signaling network involved in breast cancer brain metastases that with further research may bring forth more attractive targets.

## Methods

### Preparing astrocyte-conditioned medium (ACM)

Primary rat neonatal hippocampal astrocytes were isolated, cultured, and identified in Dr. David Harder’s laboratory who has more than 20 years of experience on it, which has been described in our previous work.^17^ In this study, isolated cells were plated at a density of ~3000 cells per mm^2^. Cells were cultured in Dulbecco’s Modified Eagle’s medium (DMEM, Gibco) with 10% fetal bovine serum (FBS). Culture media was changed three times per week. Confluent monolayers of astrocytes formed within 7–10 days after the initial plating. The primary cultures were maintained by passaging the cells when they were >90% confluent. When cell confluency was ≥70%, the complete medium was aspirated, and the cells were then incubated in serum-free medium for another 24 h. After that, the culture supernatant was collected by centrifugation to remove the cell pellets and then filtered the supernatant through a 0.45-μm filter—this final obtained medium was named astrocyte-conditioned medium (ACM). ACM was collected for experiments at passages 3–5. Primary human astrocytes were purchased from ScienCell Research Laboratories, Inc., and cultured in Complete Astrocyte Medium (with supplements, ScienCell Research Laboratories, Inc.) following the manufacturer’s instructions. At cell confluency of ≥70%, the Complete Astrocyte Medium was removed, and the cells continued to incubate in Basal Astrocyte Medium (without supplements, ScienCell Research Laboratories, Inc.) for another 24 h. hACM was collected for experiments at passages 3–5.

### Cell culture

MDA-MB-231-luc2-GFP (hereinafter referred to as MDA-MB-231) cell was purchased from Perkin Elmer. MDA-MB-468, HCC1937, MCF-7, and MCF-10A were purchased from ATCC. The MDA-MB-231 cells and MDA-MB-468 cells were cultured in DMEM with 10% FBS. HCC1937 cells were cultured in RPMI-1640 medium (Gibco) with 10% FBS. MCF-7 cells were cultured in DMEM with 0.01 mg/ml human recombinant insulin and 10% FBS. MCF-10A cells were cultured in Mammary Epithelial Cell Growth Medium (MEGM, Lonza/Clonetics Corporation) with 100 ng/ml cholera toxin and 10% FBS.

To evaluate the effect of ACM on the tumor cells, tumor cells were sequentially passaged in the ACM for indicated numbers of passages, allowing 2–3 days of growth between each passage. To evaluate whether ACM is necessary for ACM-induced tumor cell changes, MDA-MB-231 cells that had been passaged in ACM for five passages (P5A) were re-passaged in unconditioned culture media (DMEM) for another five passages (P10AD). For generating tumor cell-conditioned media (TCM), 500,000 cells were plated in 10-cm dishes in each cell line’s respective culture medium with 10% FBS, as described above, for 24 h. The complete medium was aspirated, and cells were then incubated in 6 mL of serum-free medium for 24 h. The TCM was then collected, filtered, and used for the experiments. To evaluate the effect of tumor necrosis factor alpha (TNF-α) and interleukin-1 beta (IL-1β) in the TCM on astrocytes, TCM was pre-incubated at 37 °C for 1 h with neutralizing antibodies for TNF-α (R&D Systems) and IL-1β isotype (Abcam) to neutralize the biological activity of the two cytokines.

### Microarray analysis

Cell pellets from MDA-MB-231/P5D (*n* = 3), MDA-MB-231/P5A (*n* = 3), and MDA-MB-231/P10AD (*n* = 3) were sent to Arraystar Inc., Rockville, MD for RNA isolation and microarray analysis. RNA was isolated by using TRIzol reagent (Life Technologies). Methodologies related to RNA labeling, array hybridization, and data analysis are available upon request, and are also available directly from Arraystar Inc. The gene-level profiles were analyzed for differentially expressed genes among different treated cells as above by using R-3.5 Software. The filtration was performed by comparing the gene expression levels between MDA-MB-231/P5A and MDA-MB-231/P5D, as well as MDA-MB-231/P10AD and MDA-MB-231/P5D. Differentially expressed genes were selected by using the following criteria: fold change (FC) >2.0 and a *p*-value < 0.01.

### Quantitative PCR analysis

See supplemental file.

### ELISA

See supplemental file.

### Establishment of MDA-MB-231 cell line with stable knockdown of ANGPTL4

293T cells grown to 70% confluence in DMEM were transfected with pGFP-C-shLenti vectors expressing four unique 29-mer shRNAs for human *ANGPTL4* (*ANGPTL4* human shRNA, OriGene Technologies, Inc.), and a control 29-mer scrambled shRNA cassette (Control shRNA) in the pGFP-C-shLenti Vector (OriGene Technologies, Inc.) by using TurboFectin transfection reagent (OriGene Technologies, Inc.) following the manufacturer’s instructions. The culture media was changed after 12–18 h of incubation. Two batches of the viral supernatants of 293T cells were collected 48 and 72 h after transfection and combined and then filtered to remove cellular debris. MDA-MB-231 cells with stable knockdown of *ANGPTL4* (Angptl4shRNA) and an empty vector control (ConshRNA) were established by incubation of MDA-MB-231 cells with the viral supernatant for 5 h and puromycin selection for at least 2 weeks. The transfection efficiency was monitored by positive GFP expression. Efficient shRNA-mediated knockdown of *ANGPTL4* in MDA-MB-231 cells was confirmed by qPCR. Those colonies with the best knockdown efficiency of *ANGPTL4* were used for experiments.

### In vivo experiment

In vivo experiments were performed as described before.^17^ Briefly, 6–8-week-old male CB17/SCID mice (CB17/Icr-*Prkdc*/IcrCrl, Charles Rivers, Wilmington, MA, USA) were used in this study. Mice were inoculated with 1 × 10^5^ exponential growth phase tumor cells in a 100-µL volume via left ventricular injection. Ultrasound analysis was done by using a VisualSonics Vevo 770 to visualize the injection needle to ensure left heart ventricle injection of the cells (Additional file 3: Fig. S2A). Post injection, tumor growth was checked by imaging anesthetized mice injected with 200 µl (15 mg/ml) of D-Luciferin (Xenogen Corporation) at indicated time points by using the Lumina IVIS-100 in vivo Imaging System (Xenogen Corporation) (Additional file 3: Fig. S2A). Survival endpoint was determined based on the mouse survival rate ≤30% appearing in any one of the groups. Regions of interest were created and measured as area flux, defined by radiance (photons per second per square centimeter per steradian) according to the manufacturer’s calibration (Xenogen Corporation). The MCW Animal Care and Use Committee approved all animal procedures under protocol AUA1022.

### Transient siRNA transfection

See supplemental file.

### Immunoprecipitation (IP) and immunoblot (IB)

See supplemental file. All blots were derived from the same experiment and were processed in parallel. The full, uncropped images of all blots were included in the supplemental file: Fig. S3.

### Transcriptomics analysis

Comparisons of IL-1β and TNF-α expression in different breast cancer subtypes were performed by using publicly available transcriptome data, the Cancer Genome Atlas (TCGA), in UALCAN.^48^ Microarray gene expression data from 719 human individuals representing three different molecular subtypes of breast cancer were included in this study.

### Statistical analyses

Experiments were performed in triplicate at the minimum. Average values from the triplicate experiments were used in the analyses. Data are expressed as mean ± SD. For in vitro experiments, the two-sided Student’s *t*-test was used to compare different groups. For in vivo experiments, the Kaplan–Meier curves with log-rank tests were used to compare the BM incidences between groups. Total and brain flux was compared by using the two-sided Student’s *t*-test. Log transformation was applied to variables that were skewed. All the statistical analyses were performed by using SPSS 24 (IBM) or SAS 9.4 (SAS Institute, Inc.), and a *p*-value < 0.05 was considered as statistically significant.

### Reporting summary

Further information on research design is available in the Nature Research Reporting Summary linked to this article.

## Supplementary information

Reporting Summary

Supplementary file

## Data Availability

Differentially expressed genes comparing MDA-MB-231/P5A with MDA-MB-231/P5D and MDA-MB-231/P10AD with MDA-MB-231/P5D by microarray analysis to supporting the conclusions of this article have been included in the supplementary file. The whole datasets generated and/or analyzed during the current study are available from the corresponding author upon reasonable request. All other data generated or analyzed during this study are included in this published article (and its supplementary information file).
