# Supplementary material for: Interaction of tumor cells and astrocytes promotes breast cancer brain metastases through TGF-β2/ANGPTL4 axes
Source: NPJ Precis Oncol. 2019 Oct 3;3:24. doi: 10.1038/s41698-019-0094-1 (PMC6776663; doi:10.1038/s41698-019-0094-1)
Supplement: Supplementary file 2 — Supplementary file [file 41698_2019_94_MOESM2_ESM.pdf]

Additional file 1

Supplementary Methods

Quantitative PCR analysis

Cell pellets were collected by centrifugation. Total RNAs were extracted by TRIzol reagent followed by DNase I (Life Technologies) treatment for 2 hours at 37°C. The DNA-free RNA was further purified using RNAeasy Mini kit (Qiagen). RNA concentrations were measured by Nanodrop (Thermo Scientific), followed by reverse transcription by SuperScript III (Life Sciences). Quantitative PCR was carried out with SYBR Green I in iQ5 Multicolor Real-Time PCR Detection System (Bio-Rad). The expression levels were normalized to internal GAPDH. Primers: *ANGPTL4* forward 5'-AGACACAACTCAAGGCTCAG-3′ and *ANGPTL4* reverse 5′-CTCATGGTCTAGGTGCTTGTG-3′; *GAPDH* forward 5′-GGAGCGAGATCCCTCCAAAAT-3′ and *GAPDH* reverse 5′-GGCTGTT GTCATACTTCTCATGG -3′.

ELISA

The quantification of TGF-β2, ANGPTL4, IL-1β and TNF-α proteins in cell culture supernatant and cell lysates were assessed in duplicate by ELISA using TGF-beta2 Quantikine ELISA Kit (R&D Systems), Human ANGPTL4 ELISA Kit (Raybiotech, INC.), Human IL-1 Beta PicoKine™ ELISA Kit (Boster Biological Technology); and TNF alpha (Total) Human ELISA Kit (Thermo Fisher Scientific). For supernatant, the cell culture supernatants were collected, centrifuged, and transferred into clean tubes. For cell lysate, cells were washed with cold PBS, centrifuged, and suspended in RIPA supplemented with protease inhibitor cocktail tablets and phosphatase inhibitor (Sigma-Aldrich). Concentration was determined according to the manufacturer's instructions of each kit, normalized to cell number, and expressed as pg/10^5 cells. ELISA assays were performed following the manufacturer's instructions. All experiments were carried out at least 3 times, and all results are represented as the mean ± SD.

Transient siRNA transfection

Smad3siRNA (h) and Smad4 siRNA (h) (Santa Cruz Biotechnology) consist of pools of three to five target-specific 19-25 nucleotide sequences in length, and the control siRNA consists of a scrambled sequence. siRNAs were transfected into cells according to the manufacturer's protocol. Briefly, MDA-MB-231 cells were seeded at a density of 400,000 cells in 6 well plate and cultured for 24 hours. Cells were then washed with PBS and incubated in Opti-MEM (Thermo Fisher Scientific Inc) with a complex of 30 pmol of *SMAD3*, *SMAD4*, or control siRNA, respectively, and 9µl of Lipofectamine RNAiMAX Transfection Reagent (Thermo Fisher Scientific Inc) for 16h. The medium containing siRNA-transfection reagent complexes was then aspirated and replaced with DMEM (10% FBS) for 24 hours prior to experiments.

Immunoprecipitation (IP) and immunoblot (IB*)*

For depletion of the TGF-β2 in ACM, 100 ml ACM was incubated with 2µg of anti-TGF-β2 antibody (H-6) (Santa Cruz) at 4°C for 6 hours and then with 100 µl of protein G-conjugated agarose beads at 4°C overnight. Normal mouse IgG was used as control. Upon centrifugation, the supernatant was collected to remove the immuno-complexes bound to the pelleted protein G-Sepharose, and was then used to culture tumor cells. For checking the complex of Smad3/Smad4, cell lysates were prepared by suspending cell pellets in RIPA supplemented with protease inhibitor cocktail tablets and phosphatase inhibitor. 500 µg of lysate protein was then incubated with 1µg of Smad3 (C67H9) Rabbit mAb (Cell Signaling Technology) at 4°C for 2 hours and then with 50µl of protein A-conjugated agarose beads at 4°C for 2–6 hours. Immuno-complexes bound to protein A-Sepharose were collected by centrifugation and washed with the same buffer, and then re-suspended in 2X SDS sample buffer for IB analysis. For IB, equal amounts of proteins were separated on SDS-PAGE and transferred onto polyvinylidene fluoride (PVDF) membranes (DuPont NEN). Each membrane was hybridized with primary antibody followed by horseradish peroxidase (HRP)-linked IgG and visualized by the enhanced chemiluminescence (ECL) system (Amersham). Anti-α-Tubulin Antibody (Clone DM1A, Sigma) was used for standardization of

protein loading. All experiments were repeated at least three times. All blots derive from the same experiment and were processed in parallel. Antibodies used in IB are: Anti-Smad3 (phospho S423 + S425) antibody (EP823Y) (Abcam), Smad3 (C67H9) and Smad4 (D3M6U) (Cell Signaling Technology).
